# Supplementary material for: Insights into heart failure metabolite markers through explainable machine learning
Source: Comput Struct Biotechnol J. 2025 Mar 4;27:1012–22. doi: 10.1016/j.csbj.2025.02.041 (PMC11953987; doi:10.1016/j.csbj.2025.02.041)
Supplement: MMC — From fold-specific insights to a trustworthy full-model perspective. [file mmc1.pdf]

11. Supplementary content

11.1. Training reliable models for explainability analyses

As each fold might contain fold-specific explainability profiles, here the objective was to obtain a single model explainability to filter out any potential batch effects arising from fold sample composition. We hypothesized that the explainability derived from each fold is not different from the ones of models trained on the entire dataset, suggesting that the single model explainability would be as reliable as those of the individual folds, but more practical due to its singular nature. We first confirmed that the importance scores for each fold did not differ significantly from that of the models trained on the entire dataset. Indeed, the LIME weights derived from the 5-fold cross-validation models were compared with those from the model trained on the full dataset (referred to as ”full.model”). These weights were positively correlated for both XGBoost (Pearson R = 0.68, p-value = 2.2E-16) and SVM (Pearson R = 0.79, p-value = 2.2E-16) models, indicating a high level of consistency in feature importance identified (Supplementary Figure S2). This illustrates that while some local explainability profiles vary depending on the fold composition, the feature importance in the full models is reliable, thus validating the approach of the ”full.model” explainability presented in the subsequent analyses.

12. Supplementary tables

Table S1: Dataset Overview

Dataset details	Before	After filtering and processing steps
Metabolomics Protocols	Combination of targeted mass spectrometry-based methods and gas-chromatography with flame ionization detector (GC-FID)	
Samples Origin	Plasma	
Classes	Control / Heart failure with reduced ejection fraction (HFrEF)	
Sample size	132 (Control = 72, HFrEF = 60)	124 (Control = 71, HFrEF = 53)
Measured Metabolites	71	55
Study	Asselin et al [9] - Ruiz et al [10]	

Table summarizing the metabolomics dataset used before and after quality control steps indicated in each column. Metabolomics Protocols: describes the technical methods used to obtain the metabolomics data. Samples origin: indicates the biological source of the samples. Classes: shows the two groups compared in the study: control samples and patients with heart failure. Sample size: total number of samples with a specification of the number of control samples and number HFrEF patient samples. Measured metabolites: number of metabolites. Study: cites the studies in which the data was originally reported. Abbreviations : HFrEF = heart failure with reduced ejection fraction.

Table S2: Dataset overview for the replication cohort

	Controls	HF	HFrEF
n	71	31	14
Age.Years (mean (SD))	66.04 (8.59)	70.10 (11.82)	67.86 (13.23)
Sex = 1 (%)*	42 (59.2)	14 (45.2)	9 (64.3)
NT-proBNP (mean (SD))	NaN (NA)	2053.03 (1572.08)	2712.07 (1596.78)
eGFR (mean (SD))	NaN (NA)	37.20 (10.41)	37.97 (11.85)
LVEF (mean (SD))	NaN (NA)	44.13 (14.91)	30.07 (7.92)

Table summarizing the demographic and clinical characteristics of the study samples in the replication cohort, stratified by control group (Controls), heart failure group (HF), and heart failure with reduced ejection fraction group (HFrEF). The number of participants (n) in each group is listed at the top. The control group data serve as a baseline for comparison, although NT-proBNP, eGFR, and LVEF values are not reported (NaN) for this group. The values presented in the table represent means, with standard deviations (SD) shown in parentheses. *The number and percentage of male participants in each group. Abbreviations - eGFR : estimated Glomerular Filtration Rate, FA : Fatty Acid, LVEF : Left Ventricule Ejection Fraction, NT-proBNP : N-terminal pro B-type Natriuretic Peptide.

Table S3: Performance metrics for Ridge Logistic Regression, SVM, and XGBoost computed from 5-fold cross-validation across three seeds.

Performance Metric Name	Ridge Logit	SVM	XGBoost
Seed 124			
Specificity	87.4 (77.9 – 96.9)	90.2 (80.3 – 100.0)	83.2 (78.4 – 88.0)
Sensitivity	78.0 (61.2 – 94.8)	79.8 (68.2 – 91.4)	84.6 (71.5 – 97.7)
Balanced Accuracy	82.4 (70.5 – 94.3)	84.8 (74.9 – 94.8)	84.0 (75.7 – 92.3)
Accuracy	83.4 (71.5 – 95.3)	85.8 (75.5 – 96.1)	84.0 (74.8 – 93.2)
Seed 125			
Specificity	89.0 (81.4 – 96.7)	91.8 (84.8 – 98.8)	74.0 (57.6 – 90.4)
Sensitivity	79.6 (67.8 – 91.4)	76.0 (59.6 – 92.4)	90.2 (82.4 – 98.0)
Balanced Accuracy	84.2 (75.4 – 93.0)	83.8 (76.1 – 91.5)	81.8 (72.3 – 91.3)
Accuracy	84.8 (75.9 – 93.7)	85.0 (77.7 – 92.4)	83.2 (73.9 – 92.5)
Seed 126			
Specificity	88.8 (84.0 – 93.6)	91.6 (87.7 – 95.5)	88.8 (84.0 – 93.6)
Sensitivity	77.8 (65.6 – 90.0)	79.6 (70.8 – 88.4)	85.8 (70.2 – 100.0)
Balanced Accuracy	83.2 (74.9 – 91.5)	85.4 (79.4 – 91.4)	87.2 (77.1 – 97.3)
Accuracy	83.8 (75.9 – 91.7)	86.4 (80.7 – 92.1)	87.2 (76.4 – 98.0)

Performance metrics (specificity, sensitivity, balanced accuracy, and accuracy) were obtained via 5-fold cross-validation for each seed. Values are reported as mean (95% CI), where the confidence intervals were computed based on the fold-level estimates. Since these metrics are percentages bounded between 0% and 100%, any upper CI value exceeding 100% was truncated at 100. Abbreviations: Ridge Logit = Logistic regression with a ridge penalty; SVM = Support-Vector Machine; XGBoost = eXtreme Gradient Boosting.

Table S4: Logistic Regression Results for Predicting Heart Failure (HF).

Predictor	Estimate	Std. Error	z value	p-value	OR [95% CI]
Intercept	13.48	5.40	2.50	0.013	–
C24:0	-0.38	0.10	-3.73	0.0002	0.68 [0.56 – 0.84]
Sex (Sex1)	-2.42	1.18	-2.05	0.041	0.09 [0.01 – 0.90]
Age (years)	-0.04	0.05	-0.84	0.400	0.96 [0.87 – 1.06]

OR values were obtained by exponentiating the logistic regression coefficients. The 95% confidence intervals were calculated as $\exp(\hat{\beta} \pm 1.96 \times \mathrm{SE})$. Control samples (n=71) vs HF samples (n=31) without any filter related the left ventricular ejection fraction. Abbreviations: SE = Standard Error.

Table S5: Logistic Regression Results for Predicting HF with reduced Ejection Fraction (HFrEF) status.

Predictor	Estimate	Std. Error	z value	p-value	OR [95% CI]
Intercept	13.21	5.88	2.25	0.025	–
C24:0	-0.35	0.11	-3.20	0.0014	0.70 [0.57 – 0.87]
Sex (Sex1)	-2.03	1.28	-1.58	0.113	0.13 [0.01 – 1.61]
Age (years)	-0.06	0.06	-1.07	0.283	0.94 [0.84 – 1.05]

OR values were obtained by exponentiating the logistic regression coefficients. The 95% confidence intervals were calculated as $\exp(\hat{\beta} \pm 1.96 \times \mathrm{SE})$. Control samples (n=71) vs HFrEF samples only (n=14) by using left ventricular ejection fraction (LVEF) value as filter (LVEF $\leq$ 40%) Abbreviations: SE = Standard Error.

13. Supplementary figures

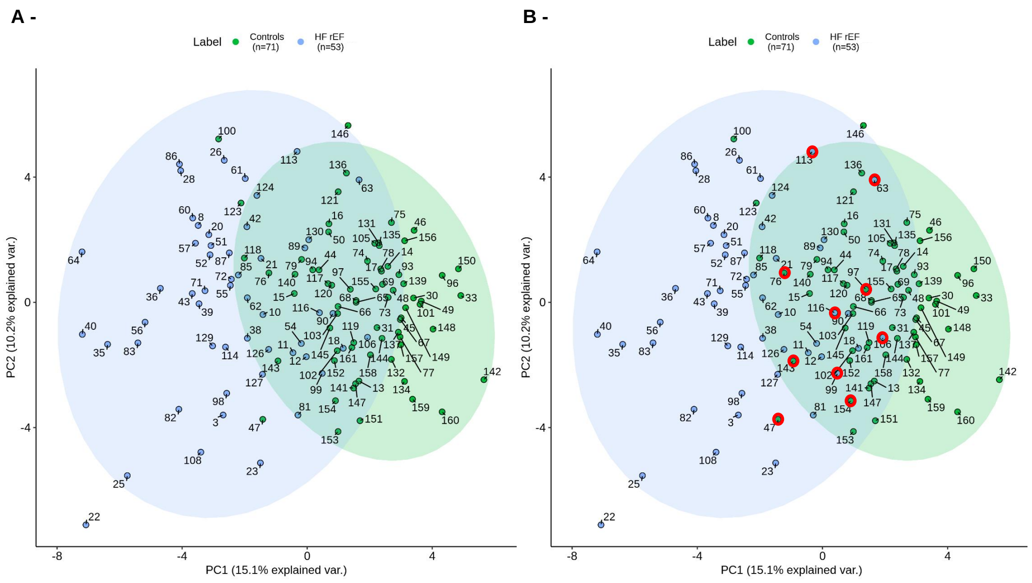

Figure S1: Principal Component Analysis (PCA) of the 124 samples and the 55 variables - Principal component analysis (PCA) of 124 samples (71 controls, 53 HF patients) using 55 variables adjusted for age and sex. Controls are identified in green, and HF patients in blue. A: Quality control using. B: PCA highlighting XGBoost misclassified samples across three seeds and 5-CV. Abbreviations: HFrEF = heart failure with reduced ejection fraction, var = variance.

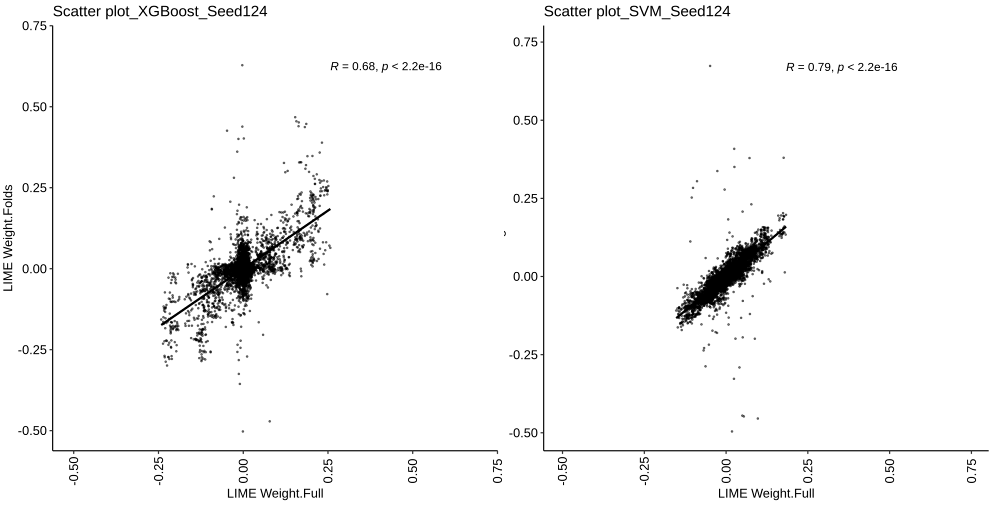

Figure S2: LIME weights comparison between 5-cross validation models and full models - Scatter plots of LIME weights. (Left) XGBoost model with seed 124. (Right) SVM model with seed 124.

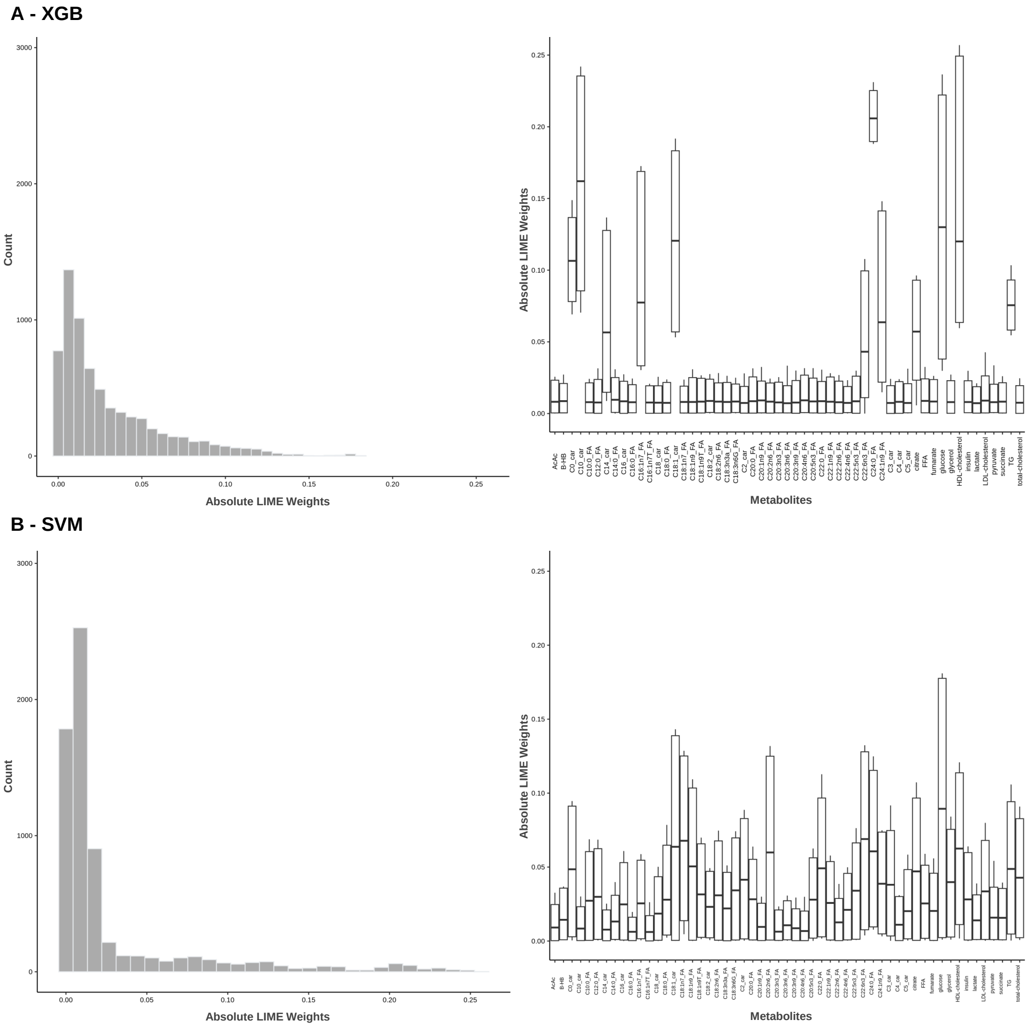

Figure S3: LIME weights distribution comparison between SVM and XGB - Total and metabolite distribution of absolute LIME weights for XGBoost (A) and SVM (B) models. Left panels - Histogram of absolute LIME weights for XGBoost, indicating the frequency of weights assigned to metabolites. Right panels - Boxplots showing the distribution of LIME weights per metabolite. Abbreviations - SVM = Support-Vector Machine , XGBoost = eXtreme Gradient Boosting, FA : Fatty Acid, car : carnitine.

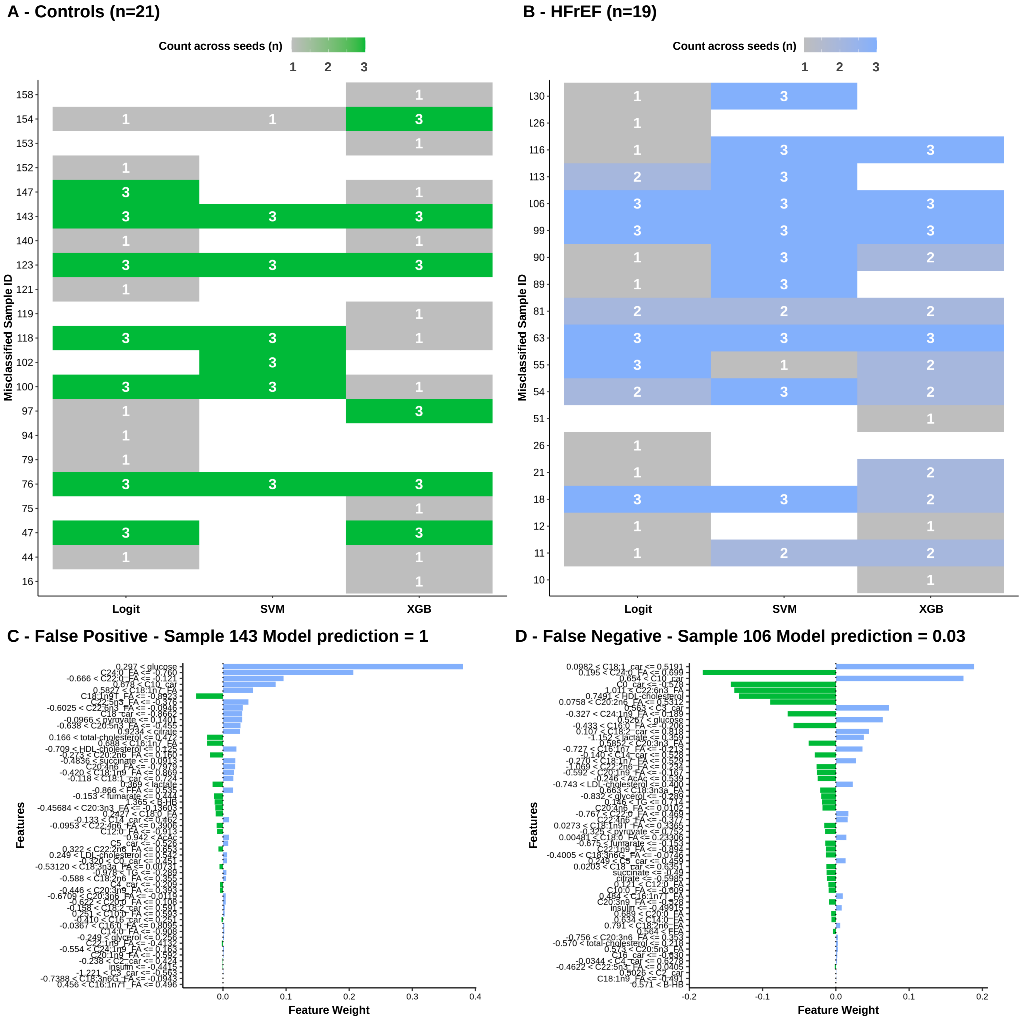

Figure S4: List of misclassified samples across various models - List of misclassified samples across Ridge Logit, SVM, XGBoost models for each seed (124,125,126). The count is representing the total occurrence of the wrong classification. The maximum possible value is 3 as each sample is tested once per seed. A: control samples. B: HFrEF samples. C: The false positive with the largest prediction error. D: The false negative with the largest prediction error.

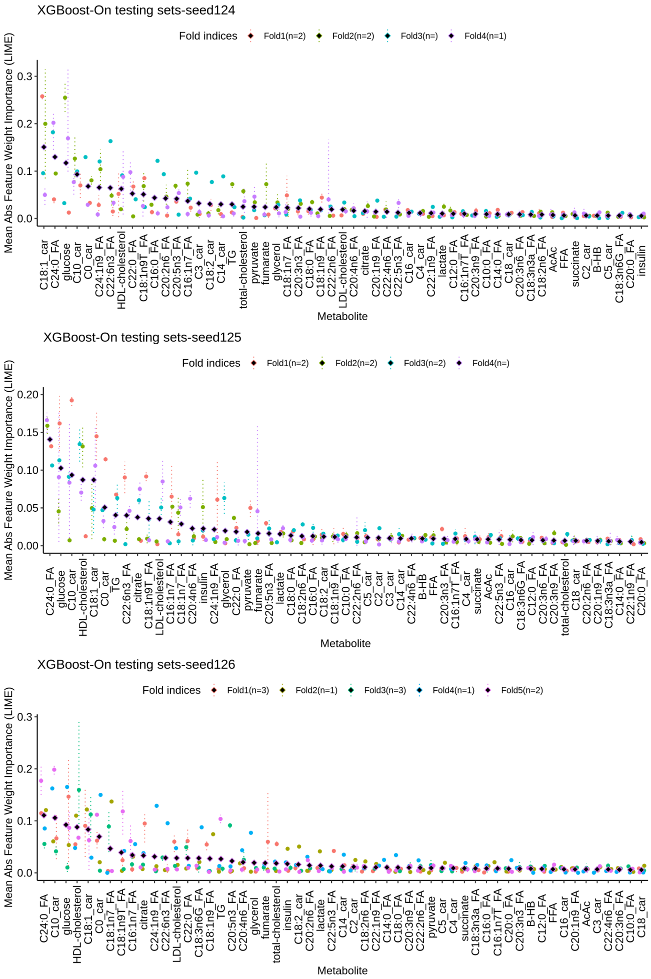

Figure S5: Wrongly classified samples for XGBoost model, important variables. - Dot plots displaying the mean absolute LIME weights for each metabolite across the folds of the XGBoost models. Each dot is color-coded according to the fold, with dotted lines indicating the minimum and maximum absolute LIME weights for the misclassified samples within a specific fold. The diamond shape represents the mean LIME weight for each metabolite across the different folds. Abbreviations used: XGBoost = eXtreme Gradient Boosting, FA = Fatty Acid, car = carnitine.

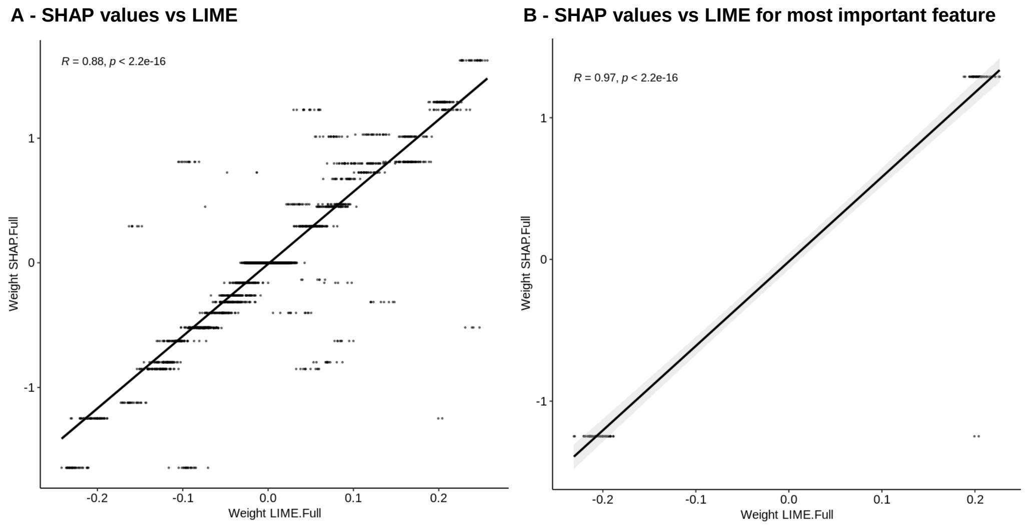

Figure S6: Local feature weights distribution comparison between SHAP values and LIME for the full model XGBoost - Scatter plots of across all samples and metabolites (A) and specifically for the most discriminant metabolite, namely C24:0_FA (B). Abbreviations - XGBoost = eXtreme Gradient Boosting, FA : Fatty Acid.
